# Association between occupational noise and vibration and anxiety in the South Korean working population: a cross-sectional study

**DOI:** 10.1186/s12995-021-00344-w

**Published:** 2022-01-03

**Authors:** Minah Park, Fatima Nari, Wonjeong Jeong, Eun-Cheol Park, Sung-In Jang

**Affiliations:** 1 Department of Public Health, Graduate School, Yonsei University, Seoul, Republic of Korea; 2 Institute of Health Services Research, Yonsei University, Seoul, Republic of Korea; 3 Department of Preventive Medicine, Yonsei University College of Medicine, 50 Yonsei-ro, Seodaemun-gu, Seoul, 03722 Republic of Korea

**Keywords:** Noise, Vibration, Anxiety, Occupation, Workplace

## Abstract

**Background:**

Although occupational exposure to noise and vibration is common, its effect on psychological wellbeing is poorly understood. This study investigated the relationship between occupational exposure to noise and vibration and anxiety among Korean workers.

**Methods:**

Data from the 5th Korean Working Conditions Survey, conducted in 2017, were used. Participants were classified into four groups according to their level of exposure, and anxiety was assessed using a self-report questionnaire. Logistic regression analysis was used to examine the significance of the association between exposure and anxiety.

**Results:**

Overall, 45,241 participants were enrolled in this study. The likelihood of anxiety increased, in both males and females, when exposed to both occupational noise and vibration (males: odds ratio (OR) = 2.25, confidence interval [CI] = 1.77–2.87; females: OR = 2.17, CI = 1.79–2.61). The association between the varying degrees of noise, vibration, and combined exposure showed a dose–response relationship among males.

**Conclusions:**

This study revealed that occupational noise and vibration exposure is associated with anxiety. These results suggest that more detailed regulations regarding occupational noise and vibration should be developed and implemented to ensure a safer environment for workers.

## Background

Anxiety is an emotion characterized by tension, worry, and physical symptoms including increased blood pressure, rapid heartbeat, and dizziness, among others [1]. According to the World Health Organization, the estimated prevalence of anxiety disorders is 3.6%, with a higher global prevalence in females (4.6%) than males (2.6%). It is estimated that 264 million people live with anxiety disorders worldwide, with a 14.9% rise in cases from 2005 to 2015 [2]. In South Korea, a 2016 survey conducted by the Ministry of Health and Welfare found that the incidence rate of an anxiety disorder was 3.8% in males and 7.5% in females [3].

High levels of occupational noise are a problem worldwide. For instance over 30 million workers in the United States of America are exposed to hazardous noise [4], whereas in South Korea, it is estimated that 2 million workers are exposed to hazardous noise [5]. Historically, occupational vibration has been overlooked. It took almost 78 years for vibration-induced injuries to be recognized as credible and deserving of compensation in the United Kingdom [6]. Many health issues and disorders are associated with exposure to vibrations, including circulatory, bone and joint, and muscle issues; neurological disorders; and disorders involving the central nervous systems [7].

Exposure to both noise and vibration can have major negative consequences. However, there is a lacuna in knowledge relating to the synergistic effect of combined noise and vibration exposure on anxiety. Further, investigation relating to occupational noise and vibration exposure can be challenging, owing to the numerous sources of extra-occupational noise and vibration [8].

Previous studies in South Korea have evaluated the relationship between noise exposure and headaches and eye strain [9]. However, the association between anxiety and exposure to noise and vibration has not been evaluated. A better understanding of this association could help in developing effective methods to prevent and manage the anxiety of workers. Thus, we investigated the association between exposure to noise and vibration and anxiety. Further, unlike previous studies that focused on the correlation between anxiety and exposure to noise and vibration individually, this study aimed to provide information about the combined effect of exposure to occupational noise and vibration.

## Methods

### Data

The data were collected from the database of the 2017 Korean Working Conditions Survey (KWCS), which was led by the Korea Occupational Safety and Health Agency. The KWCS has benchmarked the European Working Condition Survey research contents and methods and has been modified to account for cultural differences in criteria such as employment type, business type, or occupation [10]. The KWCS is used as a study to improve the working conditions in Korea, which includes categories such as quality of labor, health problems, and hazards exposure [11]. The survey included employed individuals aged 15 years and above. Face-to-face interviews were conducted through house visits across the country, using multistage systematic cluster sampling [12].

### Participants

Data were gathered from 50,205 individuals. However, after excluding those who were unable to provide information, 45,241 individuals were included in this study. Our study did not require ethical approval because the KWCS is a secondary dataset that is available in the public domain and does not contain private information.

### Variables

The dependent variable was anxiety. The KWCS asked each individual “Have you had any health problems with anxiety over the past 12 months?”, and participants were able to respond with either “Yes” or “No.”

The variables of interest were noise and vibration exposure. These were assessed using the following questions: (1) “In your workplace, are you exposed to noise so loud that you have to raise your voice to keep a conversation during work?” and (2) “How much are you exposed to hand-transmitted vibration or vibration generated by machinery?” Participants could choose one of seven responses to each question (i.e., never, almost never, one-quarter of the time, half of the time, three-quarters of the time, almost all the time, or all of the time). These were then reclassified with “never” as no exposure, and the rest of the responses were reclassified into noise exposure, vibration exposure, and noise and vibration exposure. In the subgroup analysis of our variables of interest, the degree of noise and vibration was each classified using a three-tier system. Those who answered “none” were classified as “no exposure to noise and/or vibration. The responses “almost never,” “one-quarter of the time,” “half of the time,” and “three-quarters of the time” were classified as “mild.” “Severe” included responses of “almost all the time” and “all of the time.” Noise plus vibration exposure was the combined total of the noise and vibration exposure degree and was grouped into two categories, that is, “mild” and “severe.”

The covariates included sex, age group, educational level, monthly income level, job collar, self-diagnosed presence of fatigue, hearing problems, headache/eyestrain symptoms, insomnia (the 5th edition of the Diagnostic and Statistical Manual of Mental Disorders criteria were used for diagnoses), use or non-usage of personal protective equipment, work duration, and number of working hours per week.

### Statistical analysis

A descriptive analysis was performed to examine the distribution of the general characteristics of the study population. Multiple logistic regression analysis was conducted to examine the relationship between occupational noise, vibration, and anxiety after accounting for potential confounding variables, including demographic, socioeconomic, and health-related characteristics. The results were reported using odds ratios (ORs) and 95% confidence intervals (CIs). The data were analyzed and then stratified by sex using SAS 9.4 (SAS Institute Inc.; Cary, North Carolina).

## Results

The results of the univariate analyses, which examined the association between occupational exposure to noise and/or vibration and anxiety with each variable stratified by sex, are presented in Table 1. Among the 45,241 participants, 21,612 were male, and 23,629 were female. The overall incidence rate of anxiety was higher when exposed to both noise and vibration (3.6% in males and 4.4% in females).

**Table 1:** General characteristics of Study Subjects

		Anxiety									
		Male					Female				
		Yes		No			Yes		No		
		N	%	N	%	***P*** Value	N	%	N	%	***P*** Value
Total (***n*** = 45,241)		643	(3.0)	20,969	(97.0)		761	(3.2)	22,868	(96.8)	
**Noise and vibration exposure**						<.0001					<.0001
No exposure		97	(1.6)	5812	(98.4)		171	(1.8)	9176	(98.2)	
Vibration exposure		22	(2.1)	1045	(97.9)		24	(2.4)	958	(97.6)	
Noise exposure		46	(3.5)	1285	(96.5)		74	(3.7)	1953	(96.3)	
Noise and Vibration exposure		478	(3.6)	12,827	(96.4)		492	(4.4)	10,781	(95.6)	
**Age**						<.0001					<.0001
≤39		138	(2.2)	6044	(97.8)		143	(2.5)	5609	(97.5)	
40–49		179	(3.6)	4802	(96.4)		141	(2.5)	5534	(97.5)	
50–59		186	(3.5)	5125	(96.5)		243	(3.6)	6444	(96.4)	
≥60		140	(2.7)	4998	(97.3)		234	(4.2)	5281	(95.8)	
**Education level**						0.0249					<.0001
Middle school or lower		121	(3.7)	3164	(96.3)		221	(4.3)	4922	(95.7)	
High school		218	(2.7)	7760	(97.3)		307	(3.3)	9013	(96.7)	
University and higher		304	(2.9)	10,045	(97.1)		233	(2.5)	8933	(97.5)	
**Job collar**						0.0313					<.0001
White		187	(3.0)	5995	(97.0)		166	(2.4)	6713	(97.6)	
Blue		289	(2.7)	10,355	(97.3)		218	(3.5)	6012	(96.5)	
Pink		167	(3.5)	4619	(96.5)		377	(3.6)	10,143	(96.4)	
**Income**						<.0001					<.0001
Low		128	(3.6)	3433	(96.4)		356	(3.9)	8669	(96.1)	
Mid-low		152	(2.5)	5931	(97.5)		210	(2.3)	9071	(97.7)	
Mid-high		157	(2.5)	6190	(97.5)		98	(2.8)	3406	(97.2)	
High		206	(3.7)	5415	(96.3)		97	(5.3)	1722	(94.7)	
**Fatigue**						<.0001					<.0001
Yes		483	(9.5)	4628	(90.5)		600	(10.0)	5396	(90.0)	
No		160	(1.0)	16,341	(99.0)		161	(0.9)	17,472	(99.1)	
**Hearing problem**						<.0001					<.0001
Yes		52	(13.9)	321	(86.1)		43	(14.6)	252	(85.4)	
No		591	(2.8)	20,648	(97.2)		718	(3.1)	22,616	(96.9)	
**Headache/ Eye Strain**						<.0001					<.0001
Yes		340	(12.0)	2484	(88.0)		392	(11.6)	2976	(88.4)	
No		303	(1.6)	18,485	(98.4)		369	(1.8)	19,892	(98.2)	
**Insomnia**						<.0001					<.0001
Yes		206	(6.4)	3033	(93.6)		312	(7.1)	4094	(92.9)	
No		437	(2.4)	17,936	(97.6)		449	(2.3)	18,774	(97.7)	
**Use of PPE**						0.0683					0.1485
Yes		184	(2.7)	6714	(97.3)		127	(2.9)	4291	(97.1)	
No		459	(3.1)	14,255	(96.9)		634	(3.3)	18,577	(96.7)	
**Work hour (hours)**						<.0001					<.0001
≤40		223	(2.4)	9266	(97.6)		377	(3.1)	11,832	(96.9)	
41–50		214	(3.5)	5917	(96.5)		140	(2.4)	5625	(97.6)	
51–60		120	(3.0)	3833	(97.0)		152	(3.8)	3890	(96.2)	
≥61		86	(4.2)	1953	(95.8)		92	(5.7)	1521	(94.3)	
**Work duration (years)**						0.6366					0.0002
≤5		201	(2.8)	6862	(97.2)		296	(2.9)	10,054	(97.1)	
5–10		199	(3.0)	6534	(97.0)		225	(3.1)	7072	(96.9)	
≥10		243	(3.1)	7573	(96.9)		240	(4.0)	5742	(96.0)	

The results of the logistic regression analysis examining the association between occupational exposure to noise and vibration and anxiety, for all variables stratified by sex, is presented in Table 2. Regardless of sex, participants exposed to only noise (female: OR = 1.50, CI = 1.12–2.01; male: OR = 1.55, CI = 1.06–2.27), and to both noise and vibration (female: OR = 2.17, CI = 1.79–2.61; male: OR = 2.25, CI = 1.77–2.87), showed increased odds of developing anxiety.

**Table 2:** Associations between Anxiety and Subject Demographics

Variables	Anxiety								
	Male					Female			
	OR	95% CI				OR	95% CI		
**Noise and Vibration exposure**									
No exposure	1.00					1.00			
Vibration exposure	1.31	(0.80	–	2.14)		1.51	(0.96	–	2.39)
Noise exposure	1.55	(1.06	–	2.27)		1.50	(1.12	–	2.01)
Noise and Vibration exposure	2.25	(1.77	–	2.87)		2.17	(1.79	–	2.61)
**Age**									
≤ 39	1.00					1.00			
40–49	1.52	(1.17	–	1.96)		0.74	(0.57	–	0.97)
50–59	1.40	(1.06	–	1.84)		0.92	(0.70	–	1.20)
≥ 60	0.76	(0.54	–	1.08)		0.96	(0.68	–	1.37)
**Education level**									
Middle school or lower	1.08	(0.76	–	1.55)		0.78	(0.55	–	1.12)
High school	0.83	(0.65	–	1.04)		0.93	(0.73	–	1.18)
University and higher	1.00					1.00			
**Job collar**									
White	1.00					1.00			
Blue	0.53	(0.41	–	0.70)		0.70	(0.51	–	0.95)
Pink	1.18	(0.91	–	1.53)		1.17	(0.91	–	1.50)
**Income**									
Low	1.49	(1.08	–	2.05)		0.82	(0.61	–	1.09)
Mid-low	0.93	(0.72	–	1.20)		0.52	(0.40	–	0.68)
Mid-high	0.80	(0.63	–	1.01)		0.61	(0.45	–	0.83)
High	1.00					1.00			
**Fatigue**									
Yes	7.23	(5.90	–	8.85)		8.04	(6.64	–	9.75)
No	1.00					1.00			
**Hearing problem**									
Yes	2.45	(1.73	–	3.40)		2.02	(1.38	–	2.96)
No	1.00					1.00			
**Headache/Eye strain**									
Yes	3.74	(3.12	–	4.47)		3.17	(2.69	–	3.73)
No	1.00					1.00			
**Insomnia**									
Yes	2.09	(1.73	–	2.52)		2.51	(2.13	–	2.95)
No	1.00					1.00			
**Use of PPE**									
Yes	1.00					1.00			
No	1.37	(1.12	–	1.67)		1.44	(1.17	–	1.77)
**Work hour (hours)**									
≤ 40	1.00					1.00			
41–50	1.35	(1.10	–	1.66)		0.75	(0.61	–	0.93)
51–60	1.00	(0.77	–	1.28)		1.01	(0.80	–	1.27)
≥ 61	1.22	(0.91	–	1.63)		1.23	(0.93	–	1.62)
**Work duration (years)**									
≤ 5	1.00					1.00			
5–10	0.98	(0.79	–	1.23)		0.97	(0.80	–	1.18)
≥ 10	0.78	(0.62	–	0.98)		0.89	(0.72	–	1.10)

Table 3 reports the findings of the subgroup analysis stratified by the independent variables. Male participants in the low-income group who were exposed to both noise and vibration showed higher odds of anxiety (OR:2.57 CI:1.49–3.21). Females in the pink-collar group and exposed to both noise and vibration showed higher odds of anxiety (OR = 2.56, CI = 1.95–3.35).

**Table 3:** Subgroup Analysis Stratified by Independent Variables

Variables	Anxiety												
	Noise and Vibration Exposure												
	None	Vibration				Noise				Both			
	OR	OR	95% CI			OR	95% CI			OR	95% CI		
**Male**													
**Age**													
≤ 39	1.00	2.30	(0.82	–	6.40)	1.86	(0.86	–	4.01)	2.74	(1.67	–	4.49)
40–49	1.00	0.81	(0.27	–	2.44)	1.38	(0.69	–	2.76)	1.68	(1.09	–	2.59)
50–59	1.00	1.28	(0.48	–	3.41)	1.77	(0.83	–	3.80)	2.60	(1.56	–	4.34)
≥ 60	1.00	1.46	(0.58	–	3.68)	1.31	(0.54	–	3.14)	2.16	(1.28	–	3.66)
**Job collar**													
White	1.00	1.84	(0.75	–	4.48)	1.93	(1.05	–	3.55)	2.16	(1.45	–	3.21)
Blue	1.00	0.78	(0.32	–	1.90)	0.96	(0.39	–	2.36)	2.37	(1.47	–	3.80)
Pink	1.00	2.10	(0.91	–	4.84)	1.74	(0.96	–	3.15)	2.14	(1.41	–	3.22)
**Income**													
Low	1.00	1.68	(0.62	–	4.53)	1.24	(0.47	–	3.23)	2.57	(1.49	–	4.44)
Mid-low	1.00	1.38	(0.49	–	3.87)	2.87	(1.38	–	5.99)	2.57	(1.52	–	4.37)
Mid-high	1.00	0.99	(0.32	–	3.01)	1.16	(0.53	–	2.56)	2.06	(1.26	–	3.35)
High	1.00	1.49	(0.61	–	3.65)	1.26	(0.64	–	2.49)	1.99	(1.31	–	3.01)
**Insomnia**													
Yes	1.00	1.36	(0.59	–	3.13)	2.04	(1.08	–	3.87)	1.95	(1.26	–	3.01)
No	1.00	1.46	(0.80	–	2.66)	1.57	(0.98	–	2.50)	2.47	(1.85	–	3.28)
**Use of PPE**													
Yes	1.00	0.74	(0.18	–	3.06)	2.51	(0.78	–	8.06)	3.01	(1.29	–	7.00)
No	1.00	1.66	(0.97	–	2.83)	1.45	(0.97	–	2.18)	2.19	(1.70	–	2.82)
**Female**													
**Age**													
≤ 39	1.00	3.62	(1.46	–	8.96)	0.59	(0.26	–	1.35)	2.29	(1.52	–	3.46)
40–49	1.00	1.05	(0.31	–	3.58)	1.74	(0.99	–	3.05)	1.78	(1.18	–	2.68)
50–59	1.00	1.61	(0.68	–	3.77)	1.73	(1.04	–	2.88)	2.21	(1.57	–	3.11)
≥ 60	1.00	1.21	(0.51	–	2.84)	1.76	(0.96	–	3.21)	2.48	(1.70	–	3.62)
**Job collar**													
White	1.00	1.80	(0.52	–	6.17)	1.16	(0.64	–	2.08)	1.88	(1.30	–	2.72)
Blue	1.00	1.18	(0.56	–	2.47)	0.82	(0.36	–	1.86)	1.81	(1.22	–	2.67)
Pink	1.00	1.79	(0.91	–	3.51)	1.98	(1.35	–	2.59)	2.56	(1.95	–	3.35)
**Income**													
Low	1.00	1.16	(0.57	–	2.34)	1.12	(0.69	–	1.81)	2.15	(1.63	–	2.85)
Mid-low	1.00	1.74	(0.74	–	4.05)	1.49	(0.88	–	2.50)	1.92	(1.36	–	2.72)
Mid-high	1.00	2.48	(0.78	–	7.89)	2.42	(1.14	–	5.14)	2.63	(1.53	–	4.52)
High	1.00	1.94	(0.51	–	7.27)	2.45	(1.18	–	5.40)	2.82	(1.61	–	4.93)
**Insomnia**													
Yes	1.00	2.29	(1.23	–	4.29)	1.33	(0.81	–	2.18)	2.08	(1.53	–	2.82)
No	1.00	0.81	(0.38	–	1.69)	1.70	(1.19	–	2.42)	2.34	(1.85	–	2.96)
**Use of PPE**													
Yes	1.00	2.86	(1.14	–	7.20)	1.05	(0.33	–	3.35)	3.37	(1.85	–	6.13)
No	1.00	1.29	(0.74	–	2.23)	1.52	(1.12	–	2.06)	2.05	(1.68	–	2.51)

Table 4 shows the results of a subgroup analysis indicating the degree of exposure to noise or vibration and the association with anxiety. In males, there was a dose–response relationship at all degrees of exposure. However, in females, this relationship was only significant at a mild degree of exposure.

**Table 4:** Results of Degree of Noise and/or Vibration Exposure with Anxiety

Variables	Anxiety								
	Male					Female			
	OR	95% CI				OR	95% CI		
**Degree of Vibration exposure**									
None	1.00					1.00			
Mild	1.38	(1.04	–	1.83)		1.46	(1.15	–	1.86)
Severe	1.50	(1.05	–	2.14)		1.38	(0.97	–	1.96)
**Degree of Noise exposure**									
None	1.00					1.00			
Mild	1.61	(1.20	–	2.15)		1.51	(1.18	–	1.94)
Severe	1.67	(1.12	–	2.37)		1.21	(0.83	–	1.77)
**Degree of Noise and Vibration exposure**									
None	1.00					1.00			
Mild	2.04	(1.60	–	2.60)		1.41	(1.06	–	1.88)
Severe	2.62	(1.78	–	3.86)		2.16	(1.79	–	2.60)

## Discussion

This study used data from the 5th Korean Working Conditions Survey (KWCS), to analyze the association between occupational noise and vibration exposure, and anxiety. Our findings suggest that exposure to noise, and to both noise and vibration, have a more prominent effect on anxiety than exposure to vibration alone.

Similar to previous studies [13], there was a significant positive relationship between anxiety and noise exposure. A study conducted in Egypt found that airport workers who had a higher occupational exposure to noise showed more prominent anxiety symptoms [14]. Further, this study found that combined noise and vibration had a significant influence on anxiety. This is in line with a previous study by Oldenburg [13], which showed that the occurrence of psycho-emotional strain increased significantly with exposure to noise and vibrations simultaneously. In addition, the prevalence of anxiety was higher in females than in males in our study. This is consistent with previous findings, which showed that women are more vulnerable to mental health problems than men [15]. However, the anxiety OR was higher in males than females in the noise exposure group, and the simultaneous noise and vibration exposure group. This could be owing to the fact that male participants are more likely to be exposed to hazardous occupational conditions than female participants, as reported in prior studies, and thus, the aftermath is more likely to be damaging among males [16].

Exposure to both noise and vibration can cause dysfunctions in both psychological and physical aspects linked to the nervous system [17]. Symptoms such as headache/eyestrain, fatigue, and insomnia are related to the nervous system disorders that are known to have connected mechanisms [18]. Persistent exposure can continuously agitate the autonomic nervous system, resulting in a sustained central autonomic activation and stimulation of sympathetic nervous activity. For nervous system-related symptoms, the peripheral nervous system induction could be a major risk factor [19]. As anxiety is linked with the autonomic nervous system involved in the human stress response, other symptoms could be different stressors, all contributing to the anxiety.

Our results showed higher anxiety among women in pink collar jobs. These characteristics can be attributed to job differences. In the case of females, many were working in pink-collar jobs [20]. Many pink-collar jobs are a part of the service sector, in which exposure to noise and vibration is frequent. For instance, telemarketers are continuously exposed to noise and vibration through the ringing of the phone., which could result in a higher chance of anxiety due to the work environment [21].

There was also an age difference observed between males and females. The probability of anxiety was higher among males below the age of 39. These could be owing to the fact that younger workers are likely required to undertake more difficult work compared to their seniors [22]. Additionally, previous studies have reported that well-being and mental health increases with age [23].

A dose–response relationship was seen in the variable of interest subgroup analysis, examining the relationship of exposure to noise or vibration with anxiety. Among males, the risk of anxiety increased with increased noise and vibration exposure, both individually and combined, signifying a dose–response relationship. This was observed at all levels of exposure. However, this was only observed at a milder degree of exposure among females. This may be explained by the findings of a previous study, which showed that even within the same jobs, females were less exposed to noise than males, owing to differences in tasks [24]. This is in line with the results of the current study. According to many prior studies, the synergistic effect of noise and vibration has been shown to impact on health outcomes, such as cognitive performance, hearing loss, and headache/eyestrain [25]. In animal studies, conducted by Hamernik, and in an experimental study conducted by Huang and Griffin [26], a prediction model was created for 24 healthy young people in order to examine the level of discomfort caused by exposure to continuous, whole-body vibration and noise. It was concluded that the negative psychological effects increased owing to the combination of noise and whole-body vibration, which included increased stress, discomfort, or strain [27].

This study has several limitations. First, as it is a cross-sectional survey, causalities could not be clearly confirmed. Second, the data were self-reported, and it was not possible to confirm the participants’ level of exposure, level of intensity, or the exact timeline of the noise and vibration. Owing to the question limitations, the exact decibels regarding noise exposure, or the exact amplitude, frequency, or duration regarding vibration, could not be observed. Third, the KWCS did not contain relevant information, such as drinking or smoking habits, and we were therefore unable analyze these factors. Fourthly, we could not investigate specific anxiety disorders, such as panic disorder, social phobia, and obsessive compulsive disorder [28]. It would be beneficial for further studies to include a more detailed examination of anxiety disorders in the context of occupational exposure to noise and vibrations. Fifthly, there could be different types of vibration, such as whole-body vibrations, or hand–arm vibrations, which were not considered in this study [25].

Despite its limitations, this study also has several strengths. Firstly, it used the most recent data available, which was nationally representative and collected through rigorous, systematic multistage sampling. Therefore, these results are representative of workers in Korea. Secondly, to our knowledge, this is the first study, in South Korea, to examine the relationship between exposure to noise and vibration and anxiety using KWCS data.

## Conclusions

In conclusion, this study found an association between occupational noise and vibration exposure and anxiety. Additionally, noise exposure had a bigger influence on anxiety than vibration exposure. Simultaneous noise and vibration exposure increased the likelihood of anxiety. These findings suggest that more detailed regulations are needed to manage and reduce occupational noise and vibration to provide a safer environment for workers. Additional public health measures should be put in place regarding the mental health of workers who are frequently exposed to hazardous noise and vibration.

## Data Availability

The datasets generated and/or analyzed during the current study are available in the Occupational Safety and Health Research Institute website, [https://oshri.kosha.or.kr/eoshri/resources/KWCSDownload.do].

## Abbreviations

KWCS: Korean working conditions survey
OR: Odds ratio
CI: Confidence interval
